# Risk stratification and survival prediction in heart failure: from grades to scores

**DOI:** 10.3389/fcvm.2025.1676441

**Published:** 2025-10-27

**Authors:** Huang Sidie, Zhang Wen, Zeng Yidi, Long Yun, Liang Hao

**Affiliations:** ^1^ Institute of TCM Diagnostics, Hunan University of Chinese Medicine, Changsha, Hunan, China; ^2^ Department of Geriatrics, The First Hospital of Hunan University of Chinese Medicine, Changsha, Hunan, China; ^3^ Department of Cardiology, The First Hospital of Hunan University of Chinese Medicine, Changsha, Hunan, China

**Keywords:** heart failure, risk stratification, prognosis, frailty, machine learning

## Abstract

Heart failure (HF) continues to pose a significant global health burden, necessitating accurate prognostic tools to guide patient management. This mini-review presents grading systems, frailty scales, and scoring models, followed by challenges and future directions. We traces the evolution of stratification and prognostic assessments in HF, beginning with the foundational NYHA functional classification and progressing to the advanced prognostic scores currently in use. We examine the historical significance and clinical relevance of NYHA grades, which have long been pivotal in evaluating HF severity. The review then shifts focus to contemporary prognostic scores, including the Seattle Heart Failure Model (SHFM), the Heart Failure Survival Score (HFSS), and emerging tools leveraging machine learning (ML) and big data. We explore specific challenges encountered in current clinical practice and outline future directions. By highlighting the strengths and limitations of these tools, this mini-review aims to provides a critical appraisal of stratification and scoring models for HF to inform their optimal application in clinical practice, ultimately enhancing patient care and outcomes in HF.

## 1 Introduction

Heart failure (HF) represents a critical global health challenge, affecting millions of individuals and placing substantial burdens on healthcare systems worldwide (1). Based on left ventricular ejection fraction (LVEF), HF was divided into heart failure with reduced ejection fraction (HFrEF, LVEF ≤ 40%) and heart failure with preserved ejection fraction (HFpEF, LVEF ≥ 50%) (2). Despite significant advancements in therapeutic interventions, the prognosis for patients with HF remains variable (3), underscoring the necessity for precise prognostic tools to guide clinical decision-making and enhance patient management.

Stratification and Prognostication in HF has evolved from rudimentary symptom-based classifications to sophisticated multivariate models. The New York Heart Association (NYHA) functional classification, introduced in 1928 (4), revolutionized clinical practice by categorizing patients into four grades of symptom severity. However, its subjectivity and poor correlation with objective biomarkers or mortality risk have driven demand for multidimensional risk stratification (5). Contemporary tools—such as the Seattle Heart Failure Model (SHFM) and Heart Failure Survival Score (HFSS)—integrate demographic, hemodynamic, and biomarker data to quantify individual mortality risk. More recently, machine learning (ML) algorithms harness big data to predict outcomes with unprecedented granularity. This review seeks to highlight the strengths and weaknesses of these tools, providing insights into their optimal application in clinical practice to improve patient care and outcomes in HF.

## 2 Grading approaches for stratification and prognosis of HF

For Historically, risk stratification in HF has relied on grading systems that categorize disease severity based on clinical presentation, hemodynamic status, or functional capacity. These systems, while foundational, exhibit distinct conceptual frameworks and limitations. The ACC/AHA HF staging system classifies HF into four progressive stages (A–D), focusing on disease evolution from risk factors (stage A) to refractory symptoms (stage D) (6). This approach emphasizes prevention and early intervention but lacks granularity for dynamic symptom assessment, rendering it less responsive to short-term clinical changes. In contrast, the New York Heart Association (NYHA) classification evaluates symptom severity during daily activities (classes I–IV) and remains a cornerstone for routine clinical decision-making due to its simplicity (7). However, its subjective nature introduces interobserver variability, and it poorly discriminates HF patients across the spectrum of functional impairment (5, 8).

For acute hemodynamic evaluation, the Killip/Forrester classification stratifies patients with acute myocardial infarction (MI)-induced HF into four classes based on signs of pulmonary congestion and peripheral hypoperfusion. While valuable in acute MI settings, its utility diminishes in chronic HF or non-ischemic etiologies (9). The Weber classification employs cardiopulmonary exercise testing (CPET)-derived peak oxygen consumption (VO_2_) to categorize HF into classes A–D, offering a quantitative assessment of exercise tolerance. This system excels in prognostication for advanced HF, particularly in identifying candidates for ventricular assist devices or transplantation (10). However, its reliance on CPET limits widespread applicability, especially in resource-constrained settings (11).

A comparative analysis reveals key trade-offs (Table 1). ACC/AHA staging and NYHA classification serve complementary roles—the former guiding long-term management and the latter monitoring daily symptom fluctuations. Yet both overlook comorbidities and non-cardiac contributors to prognosis. Killip/Forrester excels in acute MI but lacks relevance in chronic HF, while Weber's objectivity is counterbalanced by logistical challenges. Importantly, these systems are largely unidimensional, neglecting multidimensional risk factors such as renal function, biomarkers, or frailty, which significantly influence outcomes.

**Table 1:** Comparison of different grading approaches for stratification and prognosis of heart failure.

Systems	Parameters assessed	Key applications	Prognostic value	Strengths	Limitations
ACC/AHA stages	Risk factors, structural changes	Chronic HF risk prevention	Identifies pre-symptomatic disease	Guides preventive strategies	Static, insensitive to acute changes
NYHA class	Symptom severity	Routine clinical assessment	Quick bedside tool	Simple, widely adopted	Subjective, variable interpretation
Killip/Forrester	Hemodynamic stability	AMI with acute HF	Predicts in-hospital mortality	Rapid risk stratification	Limited to ischemic HF
WEBER classification	Exercise capacity (VO₂)	Chronic HF	Strong mortality correlation	Objective, prognostic power	Requires specialized testing

## 3 Frailty scales for stratification and prognosis of heart failure

Frailty scales have emerged as a critical advancement in HF stratification, addressing the limitations of traditional grading systems by integrating multidimensional assessments of physiological vulnerability. Unlike conventional approaches that focus narrowly on cardiac-specific metrics, frailty scales evaluate systemic functional decline, incorporating both subjective and objective measures of physical performance, cognitive status, and comorbidities (12). This holistic framework enhances risk stratification by capturing the interplay between HF severity and age-related multisystem impairments, which are strong predictors of mortality, hospitalization, and quality of life.

The Fried frailty phenotype (13) and Short Physical Performance Battery (SPPB) (14) are among the most widely validated tools. The Fried criteria define frailty as the presence of ≥3 components (unintentional weight loss, exhaustion, low physical activity, slow gait, and weak grip strength), while the SPPB quantifies lower extremity function through balance, gait speed, and chair-stand tests. Both scales demonstrate prognostic value in HF populations, with frail individuals exhibiting 2–3 times higher risks of adverse outcomes compared to non-frail counterparts (15–17). However, these tools require time-consuming physical measurements, limiting their practicality in routine clinical workflows (18). To address this, simplified questionnaires like the SARC-F (assessing strength, assistance walking, rising from a chair, climbing stairs, and falls) (19) and Clinical Frailty Scale (CFS) (20, 21) have gained traction. The SARC-F, for instance, correlates strongly with death or hospitalization in HF patients (OR 1.55, 95% CI 1.03–2.35) (22) and can be administered rapidly at bedside.

The Heart Failure Association of the European Society of Cardiology (HFA-ESC) designed a new HF frailty assessment score that encompasses four domains (23): clinical (comorbidities, weight), functional (impairment in activities of daily living, mobility and/or balance), psycho-cognitive (cognitive impairment, dementia and/or depression), and social (social support, institutionalization and/or the lack of support.) domains. A study (24) has shown that frailty assessment based on the HFA-ESC frailty domains demonstrated a high prevalence of frailty among HF patients and successfully identified individuals at elevated risk for adverse events (AUC = 0.64, 95% CI 0.60–0.68).

Recent studies highlight the superiority of frailty scales over traditional HF grading systems in identifying high-risk subgroups. For example, in the FRAIL-HF cohort, among patients hospitalized with HF, frail patients (biological phenotype criteria) showed higher 1-year all-cause mortality [HR: 2.13, 95% CI: 1.07–4.23] even after adjusting for NYHA class and ejection fraction (25). Similarly, the GUIDE-IT trial demonstrated that a higher frailty (frailty index criteria) burden was associated with a significantly higher risk of HF hospitalization or death [HR: 1.76, 95% CI: 1.20–2.58], adjusted for LVEF, NYHA class, NT-proBNP and etc. (26). These findings underscore frailty's role as a modifier of HF trajectory, particularly in aging populations with multimorbidity.

Despite their utility, frailty scales face challenges in standardization and implementation (12). Heterogeneity in assessment tools complicates cross-study comparisons, while dynamic changes in frailty status necessitate repeated evaluations. Furthermore, few scales account for HF-specific variables such as fluid retention or arrhythmia burden, which may transiently impair physical performance. Villani ER et al. (27) reported that the prevalence of frailty in AF patients ranged from 4.4%–75.4% while AF prevalence in the frail population ranged from 48.2%–75.4%. Indicators reflecting fluid retention and arrhythmia, such as edema, shortness of breath, and chest tightness, can be incorporated into the Fried frailty to provide a more comprehensive assessment of frailty in HF patients. These indicators help detect temporary functional decline caused by fluid retention or arrhythmia in HF patients, thereby enabling a more accurate evaluation of their condition. Future efforts should focus on harmonizing definitions, validating HF-tailored frailty indices, and integrating these tools into electronic health records (EHR) for automated risk alerts.

## 4 Scoring models for stratification and prognosis of HF

Risk prediction models in HF have evolved from simplistic clinical grading systems to sophisticated multivariate tools that integrate demographic, biochemical, imaging, and therapeutic data. These models aim to quantify mortality risk, guide treatment decisions, and optimize resource allocation across both chronic and acute HF populations.

### 4.1 Acute HF risk models

In the context of emergency and critical care, acute HF models are primarily designed for short-term prognostic risk stratification and optimal allocation of medical resources. These models rely on rapidly obtainable pathophysiological parameters at admission (such as blood pressure, serum creatinine, and NT-proBNP) to accurately predict in-hospital or 30-day mortality. This provides an evidence-based foundation for prioritizing triage of critically ill patients, determining eligibility for higher levels of monitoring, and guiding intensive intervention strategies during the acute phase.

For acute HF, the Emergency Heart Failure Mortality Risk Grade (EHMRG) (28, 29) and Multiple Estimation of risk based on the Emergency department Spanish Score In patients with Acute Heart Failure (MEESSI-AHF) (30, 31) emerged as frontline tools. EHMRG, validated in >12,000 emergency department patients, uses seven variables (e.g., systolic blood pressure, troponin) to predict 7-day mortality (AUC 0.79). MEESSI-AHF, incorporating NT-proBNP and potassium levels, outperforms EHMRG in 30-day risk stratification (AUC 0.85 vs. 0.80). Both models prioritize rapid risk assessment but overlook longitudinal outcomes beyond 30 days.

The GWTG-HF risk score (32) predicts in-hospital mortality using commonly available clinical variables such as age, systolic blood pressure, blood urea nitrogen, heart rate, sodium levels, chronic obstructive pulmonary disease, and non-Black race. It applies to a wide range of HF patients, including case with preserved left ventricular systolic function. Yasuyuki et al. (33) found that GWTG-HF risk score can show good discrimination and calibration in Japanese AHF patients (c-statistic, 0.763; 95% CI, 0.700–0.826), and the discriminative ability of the model was significantly improved with the addition of BNP levels (c statistic, 0.818; 95% CI, 0.771–0.865).Although the GWTG-HF score was originally developed for in-hospital patients, it also demonstrates good discrimination for 1-year mortality in a heterogeneous cohort of CICU patients (34).

### 4.2 Chronic HF risk models

The purpose of chronic HF models is to facilitate long-term risk assessment and the development of personalized advanced treatment strategies. These models integrate multidimensional variables reflecting long-term homeostasis of cardiac structure and function—such as left ventricular ejection fraction, peak oxygen consumption, and QRS duration—and are designed to predict all-cause mortality on an annualized basis. Their core clinical utility lies in providing objective, quantified criteria for patient selection and prioritization for scarce and high-risk end-stage therapies, including cardiac transplantation and left ventricular assist device (LVAD) implantation.

The HFSS (35), introduced in 1997, was among the first models to incorporate non-invasive variables—ischemic etiology, resting heart rate, left ventricular ejection fraction (LVEF), mean arterial pressure, QRS duration, serum sodium, and peak oxygen consumption (VO_2_)—to stratify heart transplant candidates. Validated in cohorts with advanced HF, HFSS demonstrated moderate discrimination (c-statistic 0.56–0.79) but faced limitations in the β-blocker era, as it excluded pharmacotherapy effects. Subsequent studies confirmed its retained prognostic value in β-blocker-treated patients, albeit with reduced sensitivity for low-risk identification (36). However, the Zugck et al. reported that HFSS was inferior to a two-variable model containing only LVEF and either peak oxygen uptake (peak VO2) or 6-min walk test (6′WT) (37).

A paradigm shift occurred in 2006 with the SHFM (38), which integrated 24 variables, including medications (*β*-blockers, ACE inhibitors) and devices (ICDs), enabling dynamic survival estimation. Derived from the PRAISE I clinical trial database and validated in 14 cohorts (*n* = 16,057), SHFM predicts 1–3-year survival with c-statistics of 0.63–0.81 (39). Its unique feature is simulating survival gains from guideline-directed therapies, such as adding sacubitril/valsartan or CRT-D. However, SHFM underestimates risk in HF with preserved ejection fraction (HFpEF) and relies on trial-derived cohorts (40, 41), limiting generalizability to real-world populations with multimorbidity. Incorporating diastolic function parameters (e.g., E/E’ ratio, IVRT) or diastolic stress biomarkers (BNP, IL-6, etc.) could enhance risk prediction accuracy, improving clinical decision-making and patient management.

The Meta-Analysis Global Group in Chronic Heart Failure (MAGGIC) score (42, 43), developed in 2013, addressed heterogeneity by pooling individual patient data from 39,372 subjects across 30 studies. This 13-variable model (e.g., age, creatinine, LVEF) predicts 1- and 3-year mortality (c-statistic 0.73) and excels in applicability across HF subtypes, including HFpEF. However, it lacks granularity in capturing acute decompensation markers or device therapy impacts.

The Metabolic Exercise test data combined with Cardiac and Kidney Indexes (MECKI) score (44), developed for chronic HF, uniquely integrates CPET parameters (e.g., VO_2_, VE/VCO_2_ slope) with renal function and LVEF. Validated in 2,715 patients, it predicts 3-year survival with superior accuracy (AUC 0.83) compared to SHFM (AUC 0.76). However, its reliance on CPET limits routine clinical application.

**Figure 1:**
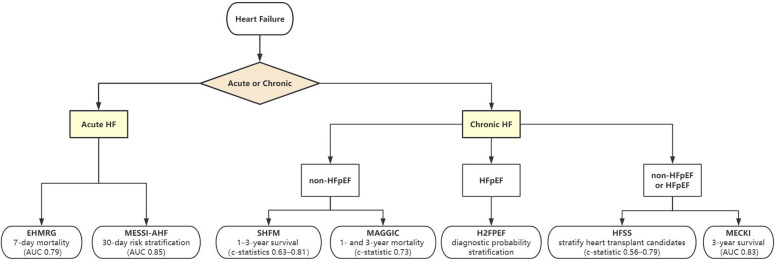
Tool selection for acute and chronic heart failure.

Figure 1 summarizes the selection of these models based on clinical context for both acute and chronic HF, while Table 2 outlines their key trade-offs. Frailty scales reflect a patient's physiological reserve and vulnerability, whereas scoring models primarily quantify cardiac-specific risk. An integrated approach that combines frailty scales with scoring models can provide a more comprehensive risk profile, thereby better guiding personalized treatment decisions. Few tools address the distinct pathophysiology of HFpEF, where SHFM and MAGGIC underperform; Discrepancies in biomarker assays (e.g., NT-proBNP vs. BNP) and EHR documentation practices hinder cross-institutional applicability; Operational inertia: Complex models like SHFM struggle with EHR integration, whereas oversimplified tools (e.g., ADHERE's 3-variable model) (45) sacrifice granularity.

**Table 2:** Comparison of different scoring models for stratification and prognosis of heart failure.

Model	Population	Derivation cohort	Variables	Endpoint	Discrimination/calibration	Exteral validation	Intended use	Key references
EHMRG	Acute HF	Patients with HF who visited the emergency departments (ED) and fulfilled the Framingham criteria for HF	Age, Transport by EMS, SBP, Heart rate, Oxygen saturation, Creatinine, Potassium, Elevated troponin level, Active cancer, Metolazone at home	7-day mortality	c-statistic = 0.803 (95% CI, 0.763 to 0.840)	c-index (7-day mortality) = 0.73 (95% CI, 0.71–0.76); c-index(30-day mortality) = 0.71 (95% CI, 0.70–0.73)	Predict acute mortality among patients with HF who present to the ED	Prediction of Heart Failure Mortality in Emergent Care, External Validation and Refinement of Emergency Heart Failure Mortality Risk Grade Risk Model in Patients With Heart Failure in the Emergency Department
MEESSI-AHF	Acute HF	Patients presenting with AHF to ED	Barthel Index score, systolic blood pres sure, age, NT-proBNP level, potassium level, elevated cardiac troponin T (cTnT) level, NYHA class IV disease, respiratory rate, low-output symptoms, oxygen saturation, episode associated with an acute coronary syndrome, left ventricular hypertro phy on electrocardiogram, and creatinine level	30-day mortality	c-statistic = 0.828 (95% CI, 0.802 to 0.853)	c-statistic = 0.80 (95% CI, 0.76 to 0.84)	Estimate individual risk for death within 30 days for patients with AHF who are admitted to the ED	Predicting 30-Day Mortality for Patients With Acute Heart Failure in the Emergency Department, External Validation of the MEESSI Acute Heart Failure Risk Score: A Cohort Study
GWTG-HF risk score	Acute HF	Data from the American Heart Association's (AHA) Get With The Guidelines-Heart Failure module (GWTG-HF)	age, systolic blood pressure, blood urea nitrogen, heart rate, sodium levels, chronic obstructive pulmonary disease, non-Black race	In-hospital mortality	c-index = 0.75	c-statistic = 0.763 (95% CI, 0.700–0.826)	Predict the risk of in hospital mortality for patients hospitalized with HF	A Validated Risk Score for In-Hospital Mortality in Patients With Heart Failure From the American Heart Association Get With the Guidelines Program, Validation of the Get With The Guideline–Heart Failure risk score in Japanese patients and the potential improvement of its discrimination ability by the inclusion of B-type natriuretic peptide level
HFSS	Chronic HF	Data on 80 clinical characteristics from 268 ambulatory patients with advanced heart failure	Ischemic cardiomyopathy, resting heart rate, LVEF, IVCD (QRS duration ≥0.12 s of any cause), mean resting blood pressure, peak V˙o2, and serum sodium	Three risk strata (high-risk stratum, medium-risk stratum, low-risk stratum)	c-statistic = 0.56–0.79	Medium risk: HR 2.65, 95% CI 1.75–4.02, *p* < 0.001; high risk: HR 5.51, 95% CI 3.64–8.33, *p* < 0.001	Stratify AHF risk of adverse outcome and select candidates for cardiac transplantation	Development and Prospective Validation of a Clinical Index to Predict Survival in Ambulatory Patients Referred for Cardiac Transplant Evaluation, Heart failure survival score continues to predict clinical outcomes in patients with heart failure receiving *β*-blockers
SHFM	Chronic HF	The PRAISE I clinical trial database	Clinical characteristics [age, gender, NYHA class, weight, LVEF, systolic blood pressure (sBP), ischemic etiology], medications (angiotensin-converting enzyme inhibitor, angiotensin receptor blocker, β-blocker, statin, aldosterone blocker, loop diuretic equivalent dose, allopurinol), device therapy (implantable cardioverter-defibrillator, cardiac resynchronization therapy) and laboratory data (lymphocyte percentage and serum sodium, hemoglobin, uric acid, total cholesterol)	Mean, 1-, 2-, and 3-year survival	c-statistics = 0.63–0.81	c-statistics = 0.75	Estimate survival of heart failure patients	The Seattle Heart Failure Model: prediction of survival in heart failure, Validation and Recalibration of Seattle Heart Failure Model in Japanese Acute Heart Failure Patients
MAGGIC score	Chronic HF	Data on 39,372 patients with HF from 30 cohort studies	Age, lower EF, NYHA class, serum creatinine, diabetes, not prescribed beta-blocker, lower systolic BP, lower body mass, time since diagnosis, current smoker, chronic obstructive pulmonary disease, male gender, not prescribed ACE-inhibitor or angiotensin-receptor blockers	1- and 3-year mortality		c-statistic = 0.73	Readily quantifies individual patient mortality risk	Predicting survival in heart failure: a risk score based on 39 372 patients from 30 studies, Heart failure in younger patients: the Meta-analysis Global Group in Chronic Heart Failure (MAGGIC)
MECKI score	Chronic HF	2,716 systolic HF patients followed in 13 Italian centers	hemoglobin, Na+, MDRD, LVEF, peak VO2 (% predicted), and VE/VCO2 slope	0%–100%	AUC values of 0.804 (0.754–0.852) at 1year, 0.789 (0.750–0.828) at 2years, 0.762 (0.726–0.799) at 3years and 0.760 (0.724–0.796) at 4years.	AUC values of 0.81 ± 0.04 (0.73–0.89) at 1year, 0.76 ± 0.04 (0.68–0.84) at 2years, 0.80 ± 0.03 (0.73–0.86) at 3years	Identify the risk of cardiovascular death + urgent heart transplant	Metabolic exercise test data combined with cardiac and kidney indexes, the MECKI score: A multiparametric approach to heart failure prognosis, The metabolic exercise test data combined with Cardiac And Kidney Indexes (MECKI) score and prognosis in heart failure. A validation study

## 5 Challenges and future prospects

Despite significant advancements in risk prediction models for HF, several challenges persist that limit their clinical utility and generalizability.

### 5.1 Limitations of prognostic models in HFpEF

Current models fail to adequately differentiate between HF subtypes, particularly HFpEF and HFrEF. Most models were derived from cohorts dominated by HFrEF patients, leading to poor calibration in HFpEF populations where distinct pathophysiological drivers such as systemic inflammation, metabolic dysregulation, and myocardial fibrosis disproportionately influence outcomes (46, 47).

Notably, most existing HFpEF-specific tools like the H2FPEF Score (48, 49) (originally designed for diagnostic probability stratification) and drug trial data (e.g., I-PRESERVE, TOPCAT) (50–53) primarily focus on diagnostic confirmation or therapeutic response rather than prognostic modeling. The HFA-PEFF score (54, 55), despite integrating echocardiographic parameters and NT-proBNP levels, still relies on static variables and fails to capture dynamic biomarker trajectories or phenotypic heterogeneity (e.g., cardiometabolic vs. elderly frail subtypes).

The modified EFFECT score (56) can be used to assess the 28-day and 1-year mortality risk in hospitalized patients with HFpEF and ADHF (AUC: 0.76 for 28-day, and 0.72 for one-year mortality). By incorporating mortality-related indicators such as age, systolic blood pressure (SBP), blood urea nitrogen (BUN), sodium, cerebrovascular disease [defined as stroke/transient ischemic attack (TIA) in ARIC], chronic obstructive pulmonary disease (COPD), and hemoglobin, it enables better identification of high-risk patients and guides clinical decision-making, including early triage, in-hospital monitoring, treatment, and early post-discharge follow-up. However, there is still a lack of external validation cohorts to confirm its generalizability.

Additionally, traditional models depend on baseline variables (e.g., LVEF, serum sodium) and neglect temporal risk modifiers such as fluctuating NT-proBNP levels with limited prognostic value in HFpEF (30% of cases show levels <125 pg/mL) (57, 58), treatment adherence patterns, or evolving comorbidities. The reliance on drug trial data further introduces selection bias, as participants often exclude HFpEF-dominant populations—such as elderly patients with multimorbidity (≥3 comorbidities in 67.4% of Asian cohorts) or underrepresented racial groups—thereby limiting real-world applicability.

### 5.2 Challenges in the AI era

**Figure 2:**
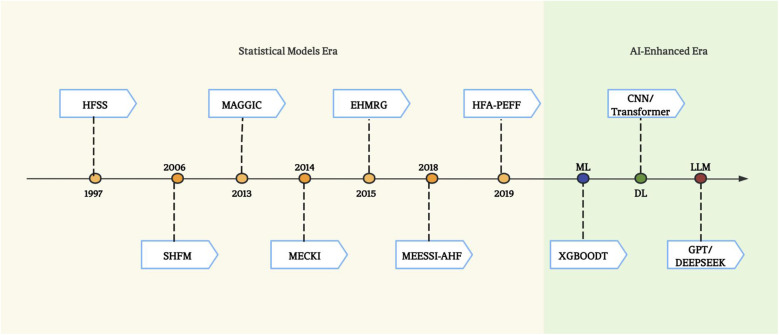
The evolution of risk stratification and survival prediction tools for heart failure.

Practical implementation barriers also hinder widespread adoption. Many scoring systems require manual data entry, which is time-consuming and prone to errors. The evolution of HF prediction into the AI era (Figure 2) with higher accuracy has witnessed three technological waves: 1) ML, have the potential to improve classification performance over traditional statistical tools by taking into account nonlinear impacts of variables to arrive at an accurate prediction (59); 2) deep learning (DL) – a branch of ML, leveraging convolutional neural networks (CNN) and recurrent neural network (RNN) for risk prediction (60, 61), outperformed traditional ML models; 3) and large language models (LLMs) capable of parsing multimodal data from EHRs and wearable devices (62).

Zhao H et al. (63) employed ML techniques such as RF and LASSO regression to construct alternative risk models. The models, which improved the accuracy of risk prediction and uncovered novel relationships between risk factors and outcomes, demonstrated good performance in predicting mortality and readmission among HFmrEF patients. Li et al. (64) developed ML algorithms to predict mortality of HF patients within ICU settings, with XGBoost demonstrating superior performance. While DL model like CNN showed a high discriminatory ability in categorizing HFpEF and control patients, achieving an AUC of 0.92 on the blinded test set, with a sensitivity of 0.98 and specificity of 0.6327 (61). HFmeRisk model, a DL model developed by Zhao X et al. (60), used both 5 clinical features and 25 DNA methylation loci to provides innovative insights into early risk assessment for HFpEF. The model underwent internal validation through tenfold cross-validation to ensure its generalization capability, and external validation with 38 samples demonstrated its reliable predictive performance (AUC = 0.82). However, due to the limited sample size, these samples may not fully represent real-world patient populations. Additionally, as the FHS cohort used in the study primarily consisted of Caucasian and a small number of East Asian individuals, the model's applicability to other ethnic groups remains unclear.

While predictive accuracy improves with model complexity—DL shows 15% or higher compared to traditional ML (65–67)—interpretability progressively declines. DL's attention mechanisms and LLM's transformer architectures create nested decision layers that obscure clinical reasoning pathways (68). For instance, transformer-based heart language models analyzing electrocardiogram reports achieved F1 score of 93.33% to detect atrial fibrillation (69), yet their self-attention weights remain clinically uninterpretable. Clinicians face a precision-transparency tradeoff: gradient boosting models reveal feature importance through SHapley Additive exPlanations (SHAP) values but fail to explain temporal models (70), while LIME (Local Interpretable Model-agnostic Explanations) provides local approximations at the cost of global coherence (71). This “black box” dilemma persists despite hybrid approaches like model distillation that compress neural networks into rule-based surrogates with 20% accuracy loss, ultimately restricting trust and routine integration. A more comprehensive understanding may be achieved by combining multiple interpretation techniques (such as integrating SHAP's global perspective with LIME's local insights). Concurrently, developing intrinsically interpretable models or designing time-series architectures optimized for explainability can embed transparency directly into the model design phase. These efforts aim to progressively bridge the “black box” dilemma and enhance clinical trust.

Some models are trained solely on data from a single institution, and their extrapolation efficacy requires further validation through multi-center studies. It is advisable to test these models using more diverse datasets from different regions and research institutions to assess their generalization capability and mitigate potential prediction biases arising from sociodemographic factors. The lack of open-source and data availability for many AI models poses a significant obstacle, as their performance can be neither independently verified nor replicated, ultimately hindering scientific progress and the widespread adoption of technology. Integration with electronic health systems or wearable devices, along with the development of mobile applications, could facilitate tighter incorporation of artificial intelligence models into clinical practice.

### 5.3 Future prospects

Future research would prioritize four key directions to address these gaps. First, HF subtype-specific models are urgently needed. HFpEF, now representing over 50% of HF cases, demands distinct predictors (e.g., atrial fibrillation burden, diastolic stress biomarkers) compared to HFrEF. Second, EHR-integrated automated scoring systems could enhance practicality by leveraging structured data (e.g., lab results, medication lists) and natural language processing to extract unstructured clinical notes. For instance, integrating SHFM variables into EHRs could enable real-time risk alerts, though this requires standardization of data formats across institutions. To further enhance data comprehensiveness, socioeconomic factors linked to survival rates—such as healthcare access or education level—can also be incorporated into the EHR. Third, multimodal data fusion—combining genomics, proteomics, and imaging-derived radiomics—may uncover novel prognostic signatures. Wearable devices enabling continuous monitoring of physiological parameters (e.g., daily step count, nocturnal heart rate variability) could further refine dynamic risk prediction. Finally, causal inference frameworks are needed to distinguish causation from correlation in longitudinal datasets, particularly when evaluating the impact of interventions like sacubitril/valsartan or cardiac resynchronization therapy.
